# Real-world experience of Dolutegravir/Lamivudine for rapid initiation of antiretroviral therapy among treatment-naïve HIV-1-infected adults in China: a multicenter retrospective study

**DOI:** 10.3389/fmed.2026.1759609

**Published:** 2026-02-11

**Authors:** Aixin Li, Xi Wang, Letian Liu, Hongwei Zhang, Zaicun Li, An Liu, Jingji Zhang, Jianwei Li, Jiangzhu Ye, Chen Chen, Hongxia Wei, Guangyong Xu, Lijun Sun

**Affiliations:** 1 Center for Infectious Diseases, Beijing Youan Hospital, Capital Medical University, Beijing, China; 2 Department of Infectious Diseases, The Second Hospital of Nanjing, Nanjing University of Chinese Medicine, Nanjing, China; 3 Department of Dermatology, Qingdao Sixth People’s Hospital, Qingdao, Shandong, China; 4 Chinese Association of STD & AIDS Prevention and Control, Care and Treatment Committee, Beijing, China

**Keywords:** antiretroviral therapy, Dolutegravir/Lamivudine, DTG/3TC, HIV, rapid ART

## Abstract

**Background:**

Experience with Dolutegravir/Lamivudine (DTG/3TC) for rapid initiation of antiretroviral therapy (ART) in newly diagnosed people living with HIV (PLWH) remains scarce. We conducted a study to evaluate the effectiveness and safety of DTG/3TC for rapid ART.

**Methods:**

This retrospective, real-world study was conducted among treatment-naïve PLWH at three centers in Beijing, Nanjing, and Qingdao. Participants were stratified into the rapid group (≤7 days) and the non-rapid group (>7 days) based on the time from HIV diagnosis to ART initiation. The primary endpoint was the rate of virological suppression (VS) at week 48, which was assessed using both intention-to-treat (ITT) and per-protocol (PP) analyses in accordance with the Food and Drug Administration (FDA) Snapshot algorithm.

**Results:**

A total of 145 participants were enrolled between February 2022 and October 2023 (57 in the rapid group and 88 in the non-rapid group). The median time for the two groups to ART initiation was 4.0 (3.0, 5.0) and 17.0 (12.3, 25.5) days, respectively (*P* < 0.001). No significant baseline differences were observed between the two groups. ITT analysis showed that the 48-week VS rates were 93.0% [95% confidence interval (CI): 86.1%–99.8%] in the rapid group and 90.9% (95% CI: 84.8%–97.0%) in the non-rapid group (*P* = 0.765). Multivariable logistic regression analysis, adjusted for age, baseline CD4 counts, baseline VL, and treatment initiation pattern, confirmed that rapid ART was not significantly associated with VS at week 48 [adjusted odds ratio (OR) = 1.100, 95% CI: 0.291–4.164, *P* = 0.888]. Subgroup analyses further demonstrated consistent results: no significant differences in VS rates were detected across subgroups (all *P* > 0.05). The median increases in CD4 counts from baseline at week 48 were 232 and 243 cells/μL in the rapid and non-rapid groups, respectively (*P* = 0.951). Throughout the 48-week follow-up period, changes in liver function, renal function, and lipid levels from baseline did not differ significantly between the two groups.

**Conclusion:**

Our study provides clinical evidence supporting the effectiveness and safety of DTG/3TC for rapid ART in treatment-naïve PLWH, with outcomes comparable to those of non-rapid initiation.

## Introduction

The global scale-up of antiretroviral therapy (ART) has transformed HIV infection into a manageable chronic condition. Nevertheless, the optimization of treatment strategies still faces some key challenges (1). In recent years, there has been an increasing trend toward adopting two-drug regimens in ART. Dolutegravir/Lamivudine (DTG/3TC) has emerged as a promising option due to its high genetic barrier, favorable safety profile, and reduced pill burden compared to traditional three-drug regimens (2). However, an evidence gap persists regarding the clinical applicability of combining rapid ART initiation with two-drug regimens. Current research has focused primarily on the efficacy, safety, and cohort retention rates of triple-drug regimens during rapid ART initiation (3). Although multiple real-world studies have confirmed that the virological suppression (VS) rate of DTG/3TC in treatment-naïve people living with HIV (PLWH) is comparable to that of three-drug regimens (4, 5), there is currently a lack of data to verify whether DTG/3TC can maintain its performance under rapid initiation of ART.

Although DTG/3TC has been recommended as a first-line ART regimen in major international and domestic guidelines (6–8), its practical implementation for rapid initiation still faces several challenges: the absence of baseline genotypic resistance testing (GRT), which may lead to unrecognized 3TC resistance (M184V mutation), and the possibility of unrecognized hepatitis B surface antigen (HBsAg) positive chronic hepatitis B infection, and the fact that some international guidelines do not recommend starting DTG/3TC in patients with a viral load (VL) > 500,000 copies/mL.

Therefore, we conducted a multicenter, real-world, retrospective study to analyze the 48-week clinical outcomes of rapid ART (≤7 days after HIV diagnosis) vs. non-rapid ART (>7 days) with DTG/3TC in Chinese PLWH, aiming to provide evidence for future clinical decision-making.

## Materials and methods

### Study design

This was a real-world, multi-center, retrospective cohort study. The study was conducted at three HIV treatment centers, namely Beijing Youan Hospital, Capital Medical University, The Second Hospital of Nanjing and Qingdao Sixth People’s Hospital. It evaluated the effectiveness and safety of treatment-naïve PLWH receiving DTG/3TC as rapid initiation of ART. According to the time of ART initiation (from the date of confirmed HIV diagnosis to the date of ART initiation), patients were divided into the rapid group (ART initiation time ≤ 7 days) and the non-rapid group (ART initiation time > 7 days).

The inclusion criteria were as follows: adults newly diagnosed with HIV-1 infection between February 1st, 2022 and October 31st, 2023, and those who received the DTG/3TC regimen as the initial treatment plan. The exclusion criteria were: (1) missing baseline data; (2) co-infection with hepatitis B virus (HBV) or estimated glomerular filtration rate (eGFR) < 30 mL/min/1.73 m^2^; (3) pregnancy; and (4) a history of allergy or high sensitivity to any components of the study drugs or auxiliary materials. Study data were collected by reviewing electronic medical records. The cut-off date for study follow-up was October 31st, 2024.

This study was approved by the Ethics Committee of Beijing Youan Hospital, Capital Medical University (No. 2022-066). The studies were conducted in accordance with the local legislation and institutional requirements. The participants provided their written informed consent to participate in this study.

### Data collection

We collected baseline demographic data (age, gender, weight), comorbidities, the time of ART initiation, and laboratory test results from the medical records. The laboratory test results included CD4 counts, human immunodeficiency virus-1 ribonucleic acid (HIV-1 RNA), GRT, liver function [alanine aminotransferase (ALT), aspartate aminotransferase (AST), gamma-glutamyl transferase (GGT), total bilirubin (TBIL), direct bilirubin (DBIL)], renal function [serum creatinine (CRE), estimated glomerular filtration rate (eGFR), cystatin C (Cys-C)], as well as random blood glucose and lipid levels [triglycerides (TG), total cholesterol (TC), high-density lipoprotein cholesterol (HDL-C), low-density lipoprotein cholesterol (LDL-C)]. All clinical evaluations were performed in accordance with the follow-up requirements of our treatment centers, and no additional testing or examination was needed.

### Definition of variables

Effectiveness: VS was defined as a plasma HIV-1 RNA < 50 copies/mL at both weeks 24 and 48, and was assessed using the Food and Drug Administration (FDA) Snapshot algorithm with two analytical approaches:

1. Intention-to-treat (ITT) analysis: This analysis included all patients enrolled in the study, regardless of their subsequent adherence to the protocol or follow-up status. Patients were classified as non-virological suppression (NVS) if they met any of the following criteria: loss to follow-up (LTFU), protocol modifications, treatment discontinuation due to adverse events, low-level viremia (LLV), or confirmed virologic failure (CVF).
2. Per-protocol (PP) analysis: This analysis was restricted to patients who completed the full follow-up period and had valid, evaluable data for all key study endpoints. NVS in this cohort was defined as the absence of VS at the predefined time points, excluding those with protocol deviations or missing data.

LLV: HIV-1 RNA levels between 50 and 200 copies/mL at the 24- and 48-week follow-up points.

Confirmed virologic failure was defined as HIV-1 RNA ≥ 200 copies/mL at the week 24 or 48 follow-up. Participants who met the CVF criteria could remain in the study, and ART was modified if treatment resistance was identified.

Antiretroviral therapy modifications were defined as patient- or doctor-requested changes to a different antiretroviral regimen for any reason (LTFU was not included in this concept). Participants with modified ART were classified as NVS but remained in the study cohort.

Initiation treatment patterns: We classified patients into three types according to the reports of VL and GRT. These three initiation treatment patterns reflect real-world variations in diagnostic completeness at ART initiation: Model 1 (VL and GRT unknown) occurs when ART is initiated before test results are available; Model 2 (VL known, GRT unknown) applies when VL results are available but GRT testing is pending; Model 3 (both VL and GRT known) indicates ART initiation after a full diagnostic workup, which is the ideal but often delayed scenario in clinical practice.

### Observation indicators

The primary endpoint was the proportion of participants with VS at 48 weeks as assessed by ITT analysis and PP analysis. The secondary endpoints included the proportion of participants with VS at 24 weeks, as well as virological effectiveness in different subgroups at 24 and 48 weeks (stratified by baseline VL, baseline CD4 counts, age, and initiation treatment patterns). In addition, we analyzed changes in CD4 counts and CD4/CD8 ratio from baseline to weeks 24 and 48, as well as changes in safety indicators (liver function, renal function, random blood glucose, and lipid levels).

### Statistical analysis

Continuous variables with abnormal distribution were analyzed using the Mann-Whitney U-test and described as the median and interquartile range (IQR). Categorical variables were expressed as numbers (percentages) and compared using Pearson’s chi-square test or Fisher’s exact test. Multivariable logistic regression was performed for the primary endpoint (VS at week 48, ITT analysis), with covariates including age, baseline CD4 counts, baseline VL, and initiation treatment patterns; 95% confidence intervals (CIs) were reported for the adjusted odds ratios (aORs) derived from this model. For all primary and secondary virological endpoints, 95% CIs were also calculated and reported to quantify the precision of effect estimates. Statistical analyses were performed using SPSS 26.0 (IBM Corp., Armonk, NY, USA) and GraphPad Prism 9.5.0 (GraphPad Software, San Diego, CA, USA). A two-tailed *P*-value < 0.05 was considered statistically significant.

## Results

### Study population

**FIGURE 1:**
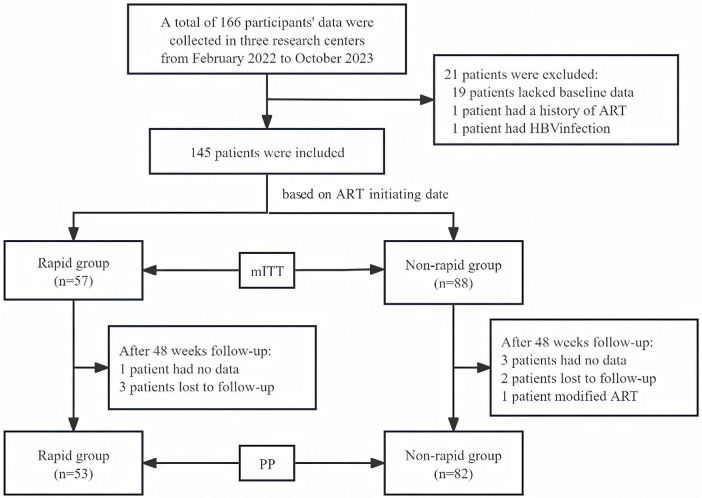
The flow chart of patient recruitment.

From February 2022 to October 2023, data from 166 participants were collected at three research centers. A total of 21 patients were excluded: 19 patients lacked baseline data, 1 patient had a history of ART, and 1 patient had HBV infection. Therefore, 145 patients were included in the study analysis pool. Among them, there were 57 cases in the rapid group and 88 cases in the non-rapid group (ITT). At the 48-week follow-up, in the rapid group, 1 participant had no data and 3 participants were lost to follow-up. Therefore, 53 participants were included in the PP analysis. In the non-rapid group, 3 participants had no data, 2 participants were lost to follow-up, and 1 participant had a protocol deviation. Eventually, 82 participants were included in the PP analysis (Figure 1).

There were 142 male and 3 female patients in this study. Ages ranged from 20 to 79 years, with a median age of 31.0 (27.0, 40.0) years, and 13.1% of participants were ≥50 years old. All patients were infected through sexual transmission, with same-sex sexual transmission among men accounting for 82.8% (120/145). Co-infections included syphilis (*n* = 49) and hepatitis C virus (HCV, *n* = 1). The median baseline CD4 count was 355 (240, 487) cells/μL, and 15.9% of participants had CD4 counts < 200 cells/μL. The median HIV-1 RNA level was 4.3 (3.7, 4.8) log_10_ copies/mL, with 18.6% having HIV-1 RNA ≥ 10^5^ copies/mL. Baseline clinical characteristics were shown in Table 1.

**TABLE 1:** Baseline characteristics of patients.

Characteristic	Total (*n* = 145)	Rapid group (*n* = 57)	Non-rapid group (*n* = 88)	*P*-value
**Demographics**				
Male, *n* (%)	142 (97.9)	55 (96.5)	87 (98.9)	0.562
Age, years, median (IQR)	31.0 (27.0, 40.0)	32.0 (28.0, 40.0)	31.0 (27.0, 40.0)	0.558
≥50 years, *n* (%)	19 (13.1)	6 (10.5)	13 (14.8)	0.616
**Co-infections, *n* (%)**				
Syphilis	49 (33.8)	20 (35.1)	29 (33.0)	0.858
HCV	1 (0.7)	1 (1.8)	0 (0)	0.393
Sexual transmission (MSM), *n* (%)	120 (82.8)	48 (84.2)	72 (81.8)	0.823
**Laboratory test**				
ALT, U/L, median (IQR)	21.0 (15.0, 31.5)	22.0 (16.5, 32.5)	19.2 (14, 29.5)	0.160
AST, U/L, median (IQR)	24.0 (21.0, 30.0)	24.0 (21.0, 29.5)	23.5 (21.0, 30.0)	0.811
GGT, U/L, median (IQR)	21.0 (16.0, 33.0)	21.0 (16.5, 33.0)	21.0 (15.0, 33.0)	0.928
TBIL, μmol/L, median (IQR)	14.2 (11.1, 19.1)	14.2 (11.1, 19.8)	14.2 (11.1, 19.1)	0.959
DBIL, μmol/L, median (IQR)	4.3 (3.4, 5.9)	4.3 (3.7, 5.7)	4.4 (3.4, 5.9)	0.767
CRE, μmol/L, median (IQR)	73.0 (65.5, 82.0)	73.0 (64.5, 81.0)	73.5 (66.0, 82.9)	0.532
eGFR, mL/min/1.73 m^2^, median (IQR)	116.2 (106.2, 121.4)	113.5 (102.3, 121.0)	118.0 (106.2, 121.6)	0.331
Cys-C, mg/L, median (IQR)	1.0 (0.9, 1.1)	1.0 (0.9, 1.1)	1.0 (0.9, 1.1)	0.892
Random blood glucose, mmol/L, median (IQR)	5.3 (4.9, 5.8)	5.4 (4.9, 5.8)	5.3 (4.8, 5.8)	0.671
TG, mmol/L, median (IQR)	1.4 (0.9, 1.9)	1.3 (0.9, 2.0)	1.4 (0.9, 1.9)	0.854
TC, mmol/L, median (IQR)	4.3 (3.9, 4.9)	4.4 (3.9, 5.0)	4.2 (3.9, 4.7)	0.203
HDL-C, mmol/L, median (IQR)	1.0 (0.9, 1.2)	1.0 (0.9, 1.2)	1.1 (0.9, 1.2)	0.847
LDL-C, mmol/L, median (IQR)	2.7 (2.3, 3.1)	2.8 (2.4, 3.4)	2.7 (2.3, 3.0)	0.085
TC/HDL-C, median, (IQR)	4.2 (3.5, 5.0)	4.3 (3.8, 4.9)	3.9 (3.4, 5.1)	0.194
HIV-1 RNA, log_10_ copies/mL, median (IQR)	4.3 (3.7, 4.8)	4.4 (3.8, 5.0)	4.1 (3.6, 4.8)	0.222
≥100,000 copies/mL, *n* (%)	27 (18.6)	13 (22.8)	14 (15.9)	0.383
CD4 counts, cells/μL, median (IQR)	355 (240, 487)	336 (244, 525)	365 (232, 481)	0.879
<200 cells/μL, *n* (%)	23 (15.9)	7 (12.3)	16 (18.2)	0.365
Baseline CD4/CD8, median (IQR)	0.3 (0.2, 0.5)	0.3 (0.2, 0.5)	0.3 (0.2, 0.6)	0.833
ART initiation date, days, median (IQR)	11.0 (4.0, 18.0)	4.0 (3.0, 5.0)	17.0 (12.3, 25.5)	<0.001
VL report duration, days, median (IQR)	8.0 (3.0, 16.0)	3.0 (0.0, 4.0)	8.0 (13.0, 23.5)	<0.001
GRT report duration, days, median (IQR)	16.0 (11.0, 23.5)	10.0 (8.0, 14.0)	21.0 (15.0, 29.8)	<0.001

Data are presented as median [interquartile range, (IQR)] or cases (percentage). IQR, interquartile range; HCV, hepatitis C virus; MSM, men who have sex with men; ALT, alanine aminotransferase; AST, aspartate aminotransferase; GGT, gamma-glutamyl transferase; TBIL, total bilirubin; DBIL, direct bilirubin; CRE, serum creatinine; eGFR, estimated glomerular filtration rate; Cys-C, cystatin C; TG, triglycerides; TC, total cholesterol; HDL-C, high-density lipoprotein cholesterol; LDL-C, low-density lipoprotein cholesterol; HIV-1 RNA, human immunodeficiency virus-1 ribonucleic acid; ART, antiretroviral therapy; VL, viral load; GRT, genotypic resistance testing.

Baseline GRT results revealed the following resistance profiles: Sixteen cases showed resistance to non-nucleoside reverse transcriptase inhibitors (NNRTIs); three cases were resistant to protease inhibitors (PIs); two cases exhibited integrase inhibitors (INSTIs) resistance, with potential resistance to elvitegravir (EVG) and raltegravir (RAL); and one case presented with concomitant resistance to both NNRTIs and PIs. Notably, no nucleoside reverse transcriptase inhibitors (NRTIs) resistance was detected in any participant included in this study.

The median time to ART initiation among the patients in this study was 11.0 (4.0, 18.0) days. In the rapid group, the median time to ART initiation was 4.0 (3.0, 5.0) days, while in the non-rapid group, it was 17.0 (12.3, 25.5) days (*P* < 0.001). The median time to report VL results was 3.0 (0.0, 4.0) days in the rapid group and 8.0 (13.0, 23.5) days in the non-rapid group (*P* < 0.001). For GRT results, the median report time in two groups were 10.0 (8.0, 14.0) days and 21.0 (15.0, 29.8) days, respectively (*P* < 0.001). There were no statistically significant differences in age, gender, liver function, renal function, random blood glucose, or blood lipids between the two groups at baseline. There were also no statistically significant differences in the median plasma HIV-1 RNA level (rapid vs. non-rapid group, 4.4 vs. 4.1 log_10_ copies/mL), the proportion of participants with HIV-1 RNA ≥ 10^5^ copies/mL (22.8% vs. 15.9%), the median CD4 count (336 vs. 365 cells/μL), and the proportion of participants with a CD4 count < 200 cells/μL (12.3% vs. 18.2%) (Table 1).

### Virological outcomes and changes in CD4 counts

According to the ITT analysis, at week 48, the VS rates in the rapid group and non-rapid group were 93.0% (53/57, 95% CI: 86.1%–99.8%) and 90.9% (80/88, 95% CI: 84.8%–97.0%), respectively (*P* = 0.765) (Table 2). Multivariable logistic regression adjusting for age, baseline CD4 counts, baseline VL, initiation treatment patterns, confirmed no significant association between rapid initiation and VS (aOR = 1.100, 95% CI: 0.291–4.164, *P* = 0.888). In the PP analysis at week 48, these rates were 100% (53/53, 95% CI: 100.0%–100.0%) and 97.6% (80/82, 95% CI: 94.2%–101.0%), respectively (*P* = 0.520) (Table 2). The VS rates of the two groups at week 24 are presented in Supplementary Table 1.

**TABLE 2:** Virological outcomes in rapid group and non-rapid group at week 48.

Virologic outcomes	Rapid group	Non-rapid group	*P*-value
ITT analysis, *n* (%)	*n* = 57	*n* = 88	**–**
VS	53 (93.0) 95% CI: 86.1%–99.8%	80 (90.9) 95% CI: 84.8%–97.0%	0.765
NVS	4 (7.0) 95% CI: 0.2%–13.9%	8 (9.1) 95% CI: 3.0%–15.2%	0.765
LLV	0 (0)	2 (2.3)	**–**
CVF	0 (0)	0 (0)	**–**
No virological data			**–**
Missing data*	1 (1.8)	3 (3.4)	**–**
LTFU	3 (5.3)	2 (2.3)	**–**
On modified ART	0 (0)	1 (1.1)	**–**
PP analysis, *n* (%)	*n* = 53	*n* = 82	**–**
VS	53 (100) 95% CI: 100.0%–100.0%	80 (97.6) 95% CI: 94.2%–101.0%	0.520
NVS	0 (0)	2 (2.4) 95% CI: −1.0%–5.9%	0.520
LLV	0 (0)	2 (2.4)	–
CVF	0 (0)	0 (0)	–

*Missing data: data was missing for this follow-up point, but participants received the study drug and the last available HIV-1 RNA < 50 copies/mL. Data are presented as cases (percentage). ITT, intention-to-treat; HIV-1 RNA, human immunodeficiency virus-1 ribonucleic acid; VS, virological suppression; NVS, non-virological suppression; LLV, low-level viremia; CVF, confirmed virologic failure; LTFU, loss to follow-up; ART, antiretroviral therapy; PP: per-protocol.

**FIGURE 2:**
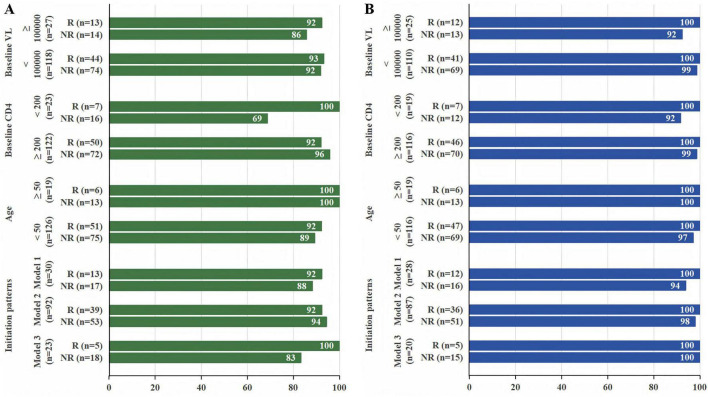
Subgroup analysis of the proportion of participants with HIV-1 RNA < 50 copies/mL **(A)** in intention-to-treat (ITT) analysis and **(B)** in per-protocol (PP) analysis at week 48. VL, viral load; R, rapid group; NR, non-rapid group.

For the subgroup analysis, based on the ITT analysis at week 48, among participants with baseline VL ≥ 10^5^ copies/mL, virologic suppression (VS) was achieved in 12/13 (92.3%, 95% CI: 75.6%–109.0%) of the rapid group and 12/14 (85.7%, 95% CI: 64.8%–106.7%) of the non-rapid group (*P* = 1.000). In participants with baseline CD4 counts < 200 cells/μL or aged ≥ 50 years, VS rates were 7/7 (100%, 95% CI: 100.0%–100.0%) vs. 11/16 (68.8%, 95% CI: 43.2%–94.3%) (*P* = 0.272) and 6/6 (100%) vs. 13/13 (100%), respectively, for the two groups (Figure 2A). Subgroup analyses for initiation patterns are also presented in Figure 2A. The PP analysis at 48 weeks showed no statistically significant differences in VS rates between the rapid group and the non-rapid group across different baseline CD4 counts, baseline VL, and age groups, as well as across the three initiation patterns (all *P* > 0.05) (Figure 2B). The VS rates of the rapid and non-rapid groups at week 24 in the subgroup analysis are presented in Supplementary Figure 1.

**FIGURE 3:**
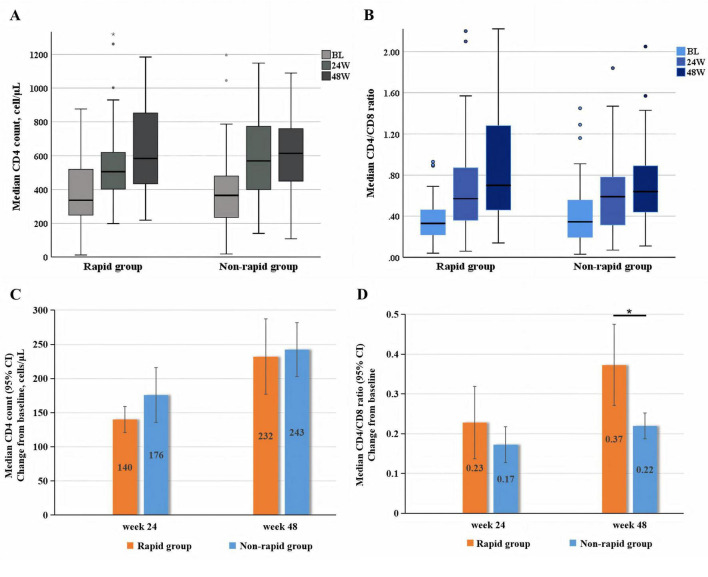
**(A)** median CD4 count and **(B)** median CD4/CD8 ratio at baseline, week 24 and week 48 in rapid group and non-rapid group. Change from baseline in panel **(C)** median CD4 count and **(D)** median CD4/CD8 ratio in rapid group and non-rapid group at week 24 and 48. *P < 0.05. BL, baseline; 24W, week 24; 48W, week 48.

At 24 weeks, median CD4 counts were 505 (400, 625) cells/μL in the rapid group and 569 (392, 789) cells/μL in the non-rapid group, both groups had significant increases from baseline (*P* < 0.05), with no between-group difference in median CD4 gain (140 vs. 176 cells/μL, *P* = 0.159). By 48 weeks, median CD4 counts rose in the rapid group and the non-rapid group were 584 (432, 859) cells/μL and 614 (445, 768) cells/μL, respectively, with marked baseline-to-week-48 elevations in both groups (*P* < 0.001) and no significant intergroup difference in median CD4 change (232 vs. 243 cells/μL, *P* = 0.951; Figure 3). Both groups also exhibited favorable immunological response in CD4/CD8 ratio at weeks 24 and 48, with significant increases from baseline at both time points (*P* < 0.001). Notably, the rapid group had a significantly greater median gain in CD4/CD8 ratio than the non-rapid group at week 48 (*P* = 0.006; Figure 3).

### Safety and tolerability

In terms of safety profile, at both 24 and 48 weeks of follow-up, there were no statistically significant differences between the rapid group and the non-rapid group in changes from baseline in liver function, renal function, random blood glucose, or blood lipids (TG, TC, HDL-C, LDL-C) (*P* > 0.05) (Table 3). However, the change in TC/HDL-C ratio from baseline was significantly lower in the rapid group than in the non-rapid group at 48 weeks (*P* = 0.024).

**TABLE 3:** Changes in laboratory results from baseline (0w) to weeks 24 and 48 in the rapid group and non-rapid group.

Indicator, median (IQR)	Changes (24w-0w)		*P*-value	Changes (48w-0w)		*P*-value
	Rapid group (*n* = 56)	Non-rapid group (*n* = 85)		Rapid group (*n* = 53)	Non-rapid group (*n* = 82)	
ALT, U/L	−2.0 (−8.5, 2.3)	−3.0 (−8.0, 4.6)	0.858	−3.0 (−11.0, 5.0)	0.0 (−0.8, 5.0)	0.693
AST, U/L	−3.0 (−5.0, 1.0)	−1.0 (−7.0, 2.4)	0.571	−3.0 (−7.0, 4.0)	−2.0 (−9.0, 1.6)	0.912
GGT, U/L	0.0 (−4.5, 5.0)	0.0 (−4.0, 4.0)	0.889	−1.0 (−7.0, 5.0)	1.0 (−4.0, 5.0)	0.201
TBIL, μmol/L	−1.2 (−3.7, 1.8)	0.0 (−3.6, 4.9)	0.227	−1.5 (−4.9, 1.4)	−0.3 (−4.8, 4.0)	0.352
DBIL, μmol/L	−0.0 (−1.1, 0.8)	−0.1 (−1.7, 1.6)	0.925	−0.1 (−1.2, 0.9)	0.0 (−1.1, 1.2)	0.530
CRE, μmol/L	7.0 (1.5, 14.0)	7.9 (2.9, 13.8)	0.605	8.0 (2.0, 14.0)	11.5 (5.0, 17.0)	0.106
eGFR, mL/min/1.73 m^2^	−6.5 (−10.6, 0.2)	−8.3 (−13.3, −2.3)	0.183	−8.7 (−13.8, -0.5)	−12.2 (−19.0, −3.2)	0.097
Cystatin C, mg/L	−0.1 (−0.1, 0.0)	−0.0 (−0.12, 0.0)	0.612	−0.1 (−0.1, −0.0)	−0.1 (−0.2, 0.0)	0.581
Random blood glucose, mmol/L	0.2 (−0.6, 0.6)	0.2 (−0.5, 0.7)	0.854	0.0 (−0.6, 0.4)	0.1 (−0.4, 0.8)	0.313
TG, mmol/L	−0.0 (−0.4, 0.4)	0.1 (−0.3, 0.6)	0.535	0.0 (−0.4, 0.7)	0.2 (−0.3, 0.6)	0.429
TC, mmol/L	0.2 (−0.5, 0.6)	0.2 (−0.2, 0.7)	0.262	0.0 (−0.4, 0.6)	0.2 (−0.3, 0.6)	0.208
HDL-C, mmol/L	0.2 (−0.0, 0.3)	0.2 (−0.0, 0.4)	0.872	0.1 (−0.1, 0.3)	0.1 (−0.1, 0.3)	0.332
LDL-C, mmol/L	0.1 (−0.2, 0.4)	0.2 (−0.2, 0.7)	0.394	0.1 (−0.2, 0.6)	0.2 (−0.2, 0.5)	0.679
TC/HDL-C	−0.4 (−1.0, −0.0)	−0.3 (−0.8, 0.2)	0.225	−0.4 (−0.9, 0.0)	−0.1 (−0.7, 0.6)	0.024

Data are presented as median (IQR). IQR: interquartile range, ALT, alanine aminotransferase; AST, aspartate aminotransferase; GGT, gamma-glutamyl transferase; TBIL, total bilirubin; DBIL, direct bilirubin; CRE, serum creatinine; eGFR, estimated glomerular filtration rate; Cys-C, cystatin C; TG, triglycerides; TC, total cholesterol; HDL-C, high-density lipoprotein cholesterol; LDL-C, low-density lipoprotein cholesterol.

No participants had laboratory serum creatinine (CRE) levels exceeding clinically relevant thresholds [CRE < 1.5 × upper limit of normal (ULN)] at week 48. Mild, transient elevations in ALT (1.5–3 × ULN) were observed in 2 (3.5%) participants in the rapid group and 2 (2.3%) in the non-rapid group, which resolved without treatment modification. In the rapid group and the non-rapid group, 5 (8.8%) and 9 (10.2%) participants were observed to have mild elevated blood lipids (1.5–2 × ULN), respectively.

During the study follow-up period, no serious adverse events (SAEs) were reported, including severe hepatotoxicity, nephrotoxicity, dyslipidemia, diabetes mellitus, neuropsychiatric disorders, osteoporosis, pathological fractures, or immune reconstitution inflammatory syndrome (IRIS). Overall, the tolerance was very good. Five patients were lost to follow-up, which was not attributed to effectiveness failure or treatment intolerance (due to relocation to another province/city). Another patient switched ART regimens after 3 months of treatment for economic reasons.

## Discussion

Up to now, there is a paucity of data evaluating the effectiveness and safety of the DTG/3TC regimen for rapid initiation of ART. This study was conducted as a real-world, multicenter, retrospective analysis to evaluate the clinical effectiveness and safety of rapid ART initiation with DTG/3TC in treatment-naïve PLWH in china, and the results are discussed in depth below by integrating our study data with the existing literature.

The primary finding of this study is that the effectiveness of the rapid group is comparable to that of the non-rapid group. This confirms the feasibility of DTG/3TC for rapid ART initiation and provides novel, high-quality clinical evidence to optimize HIV treatment strategies. Rapid ART is crucial for promptly suppressing viral replication, reducing HIV transmission, and protecting immune function (9–11), and our findings of this study support the extension of this strategy to DTG/3TC dual therapy, enriching the clinical options for rapid ART initiation.

In terms of virological effectiveness, ITT analysis revealed that at 48 weeks, the VS rate of the rapid group was 93.0%, compared with 90.9% in the non-rapid initiation group (*P* = 0.765). PP analysis demonstrated even higher VS rates (100% vs. 97.6%, *P* = 0.520), indicating that the DTG/3TC regimen maintains robust VS regardless of the time of ART initiation. This is consistent with the findings of previous real-world studies conducted in China (4, 12). After adjusting for key confounding factors including age, baseline CD4 counts, baseline VL, and initiation treatment patterns, rapid ART initiation was not significantly associated with VS at week 48 (*P* = 0.888). This indicated that the observed similarity in virological outcomes between the two groups was not confounded by baseline demographic and clinical characteristics, and verified the comparable effectiveness of rapid initiation strategy in the study cohort. Subgroup analysis further validated the robustness of this finding: in patients with high VL (≥10^5^ copies/mL) or low baseline CD4 counts (<200 cells/μL), the VS rates of the rapid group were as high as 92.3% and 100% (ITT analysis). These rates were not significantly different from those in the non-rapid group (85.7% and 68.8%) and even trended toward superiority. Similarly, the STAT study (13), a phase IIb single-arm trial, confirmed that DTG/3TC achieved a 87% VS rate at week 24 in a rapid initiation setting, even among patients with baseline VL > 1 million copies/mL. However, it is important to note that the patients enrolled in that study were initiated on ART within 14 days after diagnosis, whereas the rapid initiation threshold in our study was set at 7 days. The DOLCE study (14) evaluated the efficacy and safety of the DTG/3TC dual drug regimen in treatment-naive PLWH with CD4 counts < 200/mm^3^, confirming its efficacy in patients with advanced HIV infection. However, the interval from HIV diagnosis to ART initiation was not clearly reported in that study.

Additionally, subgroup analysis stratified by the three treatment initiation modes revealed no significant intergroup differences, further validating the adaptability of this regimen across diverse clinical practice settings. This finding aligns with the results of the D2ARLING study (15), in which the DTG + 3TC dual regimen was administered to treatment-naive PLWH without baseline GRT at ART initiation, and the 48-week VS rate was non-inferior to that achieved with the standard triple-drug regimen. This outcome holds particular clinical value for resource-limited settings or regions where HIV-1 RNA assays and GRT are not readily available, as it confirms that rapid ART initiation with the DTG/3TC regimen can be implemented without awaiting the results of such tests, thereby ensuring the timely delivery of treatment to eligible patients.

In terms of immunological recovery, CD4 counts and CD4/CD8 ratio are key indicators for evaluating immune function restoration in PLWH. Our study found that CD4 counts in both groups increased significantly at week 48, with no significant difference in the magnitude of elevation between the two cohorts, indicating that rapid ART initiation with the DTG/3TC regimen does not exert an adverse effect on immune reconstitution. Consistent with prior investigations, the magnitude of CD4 counts increase in treatment-naïve PLWH receiving DTG/3TC dual therapy was comparable to that observed with third-drug regimens based on second-generation INSTIs, thus verifying the non-inferiority of this dual therapy in terms of immune recovery (16). As widely recognized, the interval from HIV diagnosis to ART initiation constitutes a major factor influencing the CD4/CD8 ratio (17–19). In our study, we also observed a more pronounced increase in the CD4/CD8 ratio in the rapid group at week 48 compared with the non-rapid group, suggesting that rapid ART initiation with DTG/3TC may facilitate more prompt restoration of immune system homeostasis and holds important implications for improving long-term prognosis.

In terms of safety profiles, neither group exhibited significant alterations in liver function, renal function, blood glucose, or lipid levels from baseline to week 48, with no significant intergroup differences noted. These findings demonstrate that the DTG/3TC regimen possesses favorable safety characteristics when implemented for rapid ART initiation. Consistent with reports that DTG-based regimens maintain a stable TC/HDL-C ratio (16), this study further found that the TC/HDL-C ratio was more stable in the rapid group, implying that rapid initiation of DTG/3TC may potentially help reduce the risk of cardiovascular diseases in PLWH. Furthermore, no SAEs were documented throughout the study period, and patient treatment adherence was favorable. This is consistent with previous findings that the DTG/3TC regimen sustains high long-term adherence and durable virological control (20–22), thereby further corroborating the excellent safety and tolerability of DTG/3TC when administered as part of a rapid ART initiation strategy.

To date, major international and domestic guidelines for HIV treatment have not explicitly recommended the DTG/3TC regimen for rapid ART initiation. Plausible reasons for this gap include insufficient clinical evidence supporting the use of DTG/3TC within rapid initiation protocols, the lack of validation from large-scale multicenter studies, and lingering concerns regarding potential safety and efficacy risks. The findings of our study provide a robust response to these unresolved questions, filling the clinical evidence gap surrounding DTG/3TC-based rapid ART initiation through a multicenter retrospective analysis. Firstly, our results confirm that rapid ART initiation with DTG/3TC yields comparable virological effectiveness and favorable safety profiles relative to non-rapid initiation strategies, thereby supplementing high-quality real-world evidence for the applicability of DTG/3TC in rapid ART initiation settings. Secondly, no cases of baseline NRTI resistance or VL exceeding 500,000 copies/mL were identified among the enrolled participants. Although this observation might imply a lower-than-expected real-world prevalence of these theoretical contraindications, the more plausible interpretations are attributable to the predefined exclusion criteria in our study protocol and the inherent limitations of our sample size. While HBV-coinfected patients were excluded from the current analysis, screening for HBsAg should still be incorporated into rapid initiation workflows— a step that does not impede the timely implementation of ART. Additionally, no treatment discontinuations attributable to poor adherence were reported during the study period; however, intensified adherence counseling remains a critical component of care for PLWH undergoing rapid ART initiation. It is anticipated that with the accumulation of additional real-world data and prospective clinical trial evidence, future HIV treatment guidelines will incorporate targeted recommendations for DTG/3TC-based rapid ART initiation, thereby expanding the regimen’s clinical application scope and advancing the optimization of global rapid ART initiation strategies.

Despite providing valuable clinical evidence, this study has several inherent limitations that should be acknowledged: First, the retrospective, non-randomized study design may introduce residual confounding, even after multivariable adjustment. Additionally, 11.5% (19/166) of patients were excluded due to missing baseline data, which could potentially introduce selection bias. Second, the study cohort was predominantly male (97.9%), with a high proportion of men who have sex with men (MSM). The small number of female participants (*n* = 3) and older patients (≥50 years, *n* = 19) limits the generalizability of our findings to these underrepresented populations. Third, patients with hepatitis B virus (HBV) coinfection and those with an estimated glomerular filtration rate (eGFR) < 30 mL/min/1.73 m^2^ were excluded from the analysis. This exclusion restricts the generalizability of the results to individuals with major contraindications to the DTG/3TC regimen. Fourth, the 48-week follow-up duration is insufficient to evaluate long-term outcomes, such as sustained virological suppression, the development of drug resistance, and late-onset adverse events. Fifth, subgroup analyses were exploratory in nature. Given the small sample sizes of some subgroups, these analyses were underpowered, and no correction for multiple comparisons was applied. Therefore, the results of these subgroup analyses should be interpreted with caution and regarded as hypothesis-generating rather than definitive evidence. Sixth, the generalizability of our findings may be limited to clinical settings with low baseline NRTI resistance prevalence (no NRTI resistance was detected in this study) and high treatment adherence. In regions with higher GRT prevalence or suboptimal treatment adherence, the effectiveness of DTG/3TC for rapid ART initiation may require further verification. Finally, no patients in our cohort had a baseline VL > 500,000 copies/mL. Consequently, our study is unable to address the cautionary notes in current guidelines regarding DTG/3TC use in this specific high VL subgroup. Additional research involving patients with high baseline VL is therefore needed to clarify the regimen’s performance in this population. Future studies should address these limitations to provide more robust evidence for the clinical application of DTG/3TC in rapid ART initiation.

## Conclusion

In summary, this study has confirmed the effectiveness and safety of DTG/3TC in rapid initiation of ART, thereby providing evidence to support its widespread application in PLWH.

## Data Availability

The raw data supporting the conclusions of this article will be made available by the authors, without undue reservation.
