# Supplementary material for: Real-world experience of Dolutegravir/Lamivudine for rapid initiation of antiretroviral therapy among treatment-naïve HIV-1-infected adults in China: a multicenter retrospective study
Source: Front Med (Lausanne). 2026 Feb 11;13:1759609. doi: 10.3389/fmed.2026.1759609 (PMC12932182; doi:10.3389/fmed.2026.1759609)
Supplement: Supplementary file 1 [file Data_Sheet_1.docx]

**Supplementary Table 1 Virological outcomes in rapid group and non-rapid group at week 24.**

| **Virologic outcomes** | **Rapid Group** | **Non-Rapid Group** | **P-value** |
| --- | --- | --- | --- |
| **ITT analysis, n (%)** | **n=57** | **n=88** |  |
| VS | 55 (96.5)  95% CI: 91.6% – 101.4% | 81 (92.0)  95% CI: 86.3% – 97.8% | 0.483 |
| NVS | 2 (3.5)  95% CI: -1.4% – 8.4% | 7 (8.0)  95% CI: 2.2% – 13.7% | 0.483 |
| LLV | 1 (1.8) | 4 (4.5) |  |
| CVF | 0 (0) | 0 (0) |  |
| No virological data |  |  |  |
| Missing data^*^ | 0 (0) | 1 (1.1) |  |
| LTFU | 1 (1.8) | 1 (1.1) |  |
| On modified ART | 0 (0) | 1 (1.1) |  |
| **PP analysis, n (%)** | **n=56** | **n=85** |  |
| VS | 55 (98.2)  95% CI: 94.6% – 101.8% | 81 (95.3)  95% CI: 90.7% – 99.9% | 0.648 |
| NVS | 1 (1.8)  95% CI: -1.8% – 5.4% | 4 (4.7)  95% CI: 0.1% – 9.3% | 0.648 |
| LLV | 1 (1.8) | 4 (4.7) |  |
| CVF | 0 (0) | 0 (0) |  |

*Missing data: Data was missing for this follow-up point, but participants received the study drug and the last available HIV-1 RNA < 50 copies/mL.

Data are presented as cases (percentage).

ITT, intention-to-treat; HIV-1 RNA, human immunodeficiency virus-1 ribonucleic acid; VS, virological suppression; NVS, non-virological suppression; LLV, low-level viremia; CVF, confirmed virologic failure; LTFU, loss to follow-up; ART, antiretroviral therapy; PP: per-protocol.


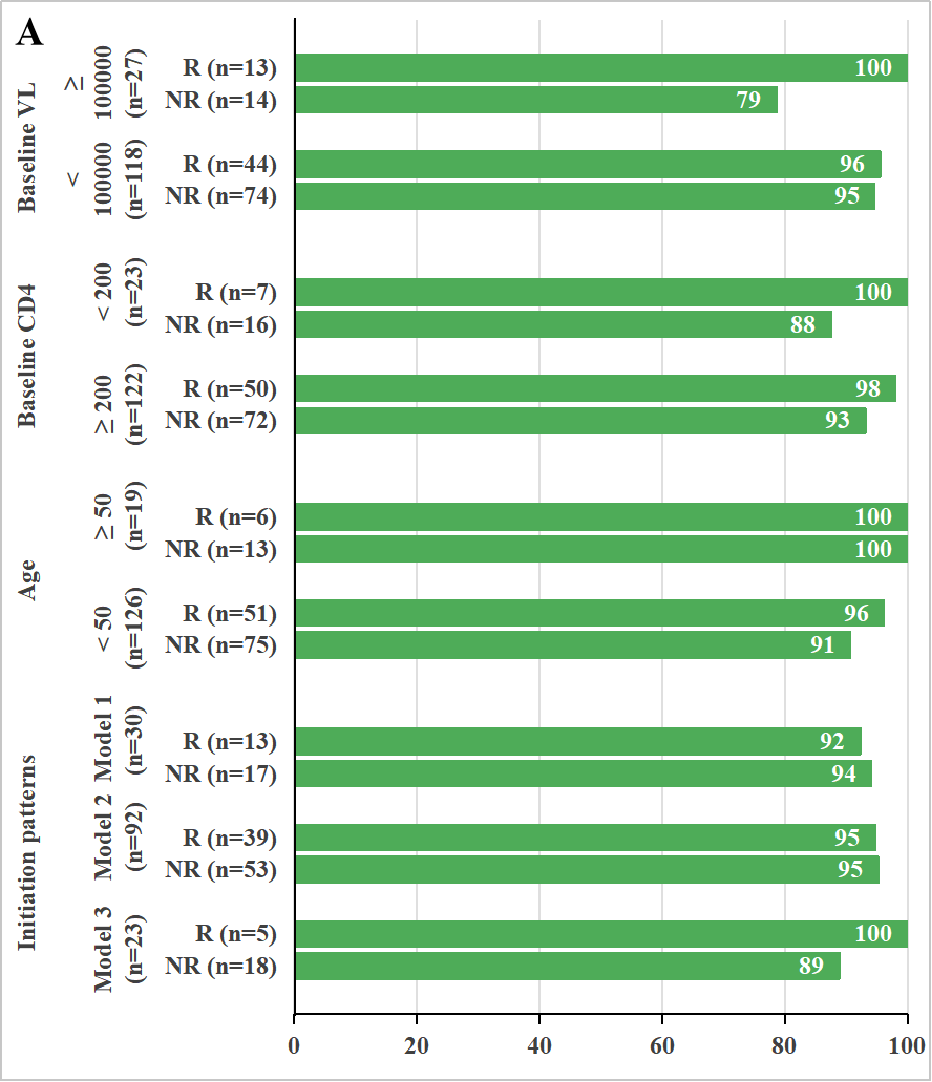

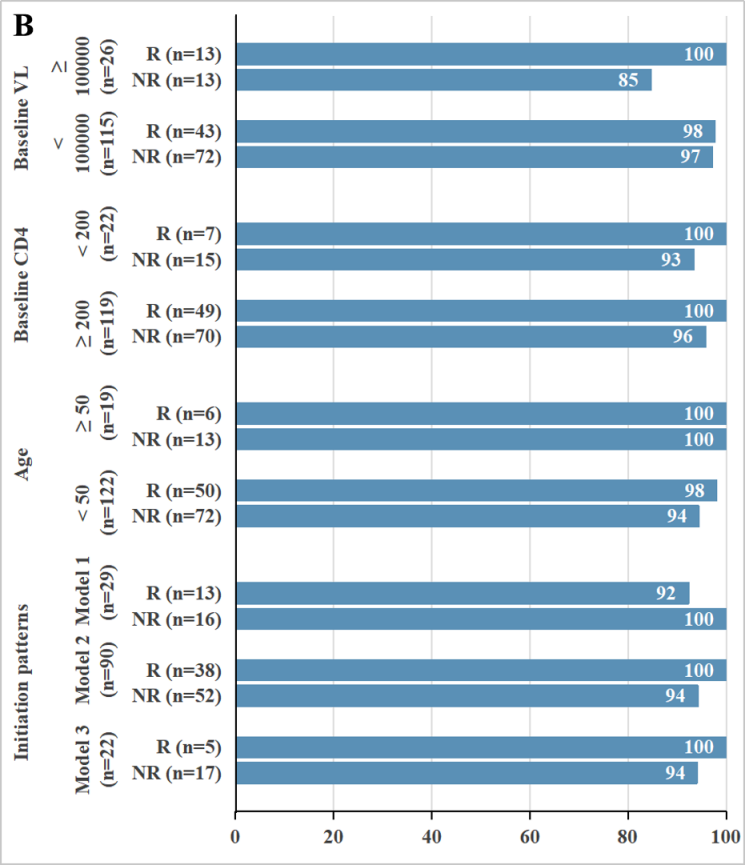


**Supplementary Figure 1**

**Subgroup analysis of the proportion of participants with HIV-1 RNA < 50 copies/mL (A) in ITT analysis and (B) in PP analysis at week 24.**

VL, viral load; R, rapid group; NR, non-rapid Group.
